# Modern management of genitourinary syndrome of menopause

**DOI:** 10.12703/r/10-25

**Published:** 2021-03-03

**Authors:** Ana Sofia Da Silva, Georgina Baines, George Araklitis, Dudley Robinson, Linda Cardozo

**Affiliations:** 1 Department of Urogynaecology, King’s College Hospital, London, UK

**Keywords:** Genitourinary syndrome of menopause, GSM, Lubricants, Moisturisers, Hyaluronic acid, Laser, Er:YAG, CO2 laser, Oestrogen, Vagifem, Ovestin, Orasterone, DHEA, Intrarosa, SERMS, Ospemifene, Osphena

## Abstract

The genitourinary syndrome of menopause (GSM) is the accepted term used to describe the broad spectrum of genitourinary tract symptoms and signs caused by the loss of endogenous sex steroids that occurs at the time of and after the menopause. Global improvements in healthcare have resulted in an ageing population. Today, women are spending 40% of their lives in the postmenopausal state, and with 50–70% of postmenopausal women reporting symptomatic GSM, safe and efficacious treatments are needed for this troublesome condition. This article reviews current evidence for non-pharmacological and pharmacological treatments with a focus on novel and minimally invasive procedures such as energy-based devices (CO_2_ laser, YAG laser), hyaluronic acid, dehydroepiandrosterone, and selective oestrogen receptor modulators.

## Introduction

Throughout the world, populations are ageing. Today, women are spending 40% of their lives in the postmenopausal state^1^. The loss of endogenous sex steroids that occurs at the time of menopause results in a profound physiological and anatomical alteration to a woman’s genitourinary system. Genitourinary syndrome of menopause (GSM) is the accepted term used to describe the broad spectrum of symptoms and signs that pertain to these changes^2^. The common clinical manifestations are summarised in Table 1. It has been reported that 50–70% of postmenopausal women have symptomatic GSM to at least some degree; however, GSM remains extremely underdiagnosed despite its high prevalence^3^.

**Table 1.** Clinical features of genitourinary syndrome of menopause (adapted from 5–7).

	Genital	Sexual	Urinary
**Symptoms**	• Vaginal dryness (most common & troublesome) • Itching/burning/irritation • Vaginal/pelvic pain and pressure	• Dyspareunia • Reduced lubrication • Loss of libido/arousal • Post-coital bleeding	• Dysuria • Urgency • Stress/urgency incontinence • Urinary tract infections • Urinary frequency/nocturia
**Signs**	• Labial atrophy • Decreased moisture • Loss of vaginal rugae • Vaginal pallor • Decreased elasticity • Higher vaginal pH level • Leukorrhea • Introital stenosis • Pelvic organ prolapse • Thinning/greying pubic hair		• Urethral prolapse/caruncle • Ischaemia of vesical trigone • Meatal stenosis

The term GSM was first introduced in 2014 following the convention for the review of nomenclature by the International Society for the Study of Women’s Sexual Health (ISSWH) and North American Menopause Society (NAMS)^2^. The terms vulvovaginal atrophy (VVA), atrophic vaginitis, or urogenital atrophy were previously used; however, the older terms were found to be insufficient to define the complexity of menopausal-associated symptoms and signs and their endocrinological impact. The previous terminology made no reference to lower urinary tract symptoms such as urgency, frequency, nocturia, and recurrent urinary tract infections; furthermore, from the public standpoint, the term “atrophy” carries negative connotations and acts as a barrier because some women are embarrassed and reluctant to use the words “vulva” or “vagina”. That notwithstanding, the new endorsed term has faced some criticisms over the labelling of a natural change as a “syndrome”^4^.

The sequelae of GSM symptoms can have a detrimental effect on a woman’s quality of life, and with an ageing population, this is likely to amplify. Contrary to vasomotor symptoms (VMS), which tend to become milder over time, symptoms of GSM are progressive and tend to deteriorate if left untreated and rarely resolve spontaneously. Consequently, they appear to have a greater impact on the sexual functioning and emotional wellbeing of affected women^5^. This article will review the latest evidence on the treatment options for GSM with a particular focus on novel agents and potential future treatment options. Throughout this review, the terms GSM, VMS, and VVA will be used, where appropriate, to remain consistent with the original language in the clinical studies and drug licensing agreements.

## Non-pharmacological treatment

### Lifestyle changes and physical therapy

The expectation of the next generation surrounding healthcare is changing; women are seeking personalised solutions and expect healthcare to be holistic^6,7^. When a woman presents with urogenital symptoms of the menopause, a detailed history should be taken to establish any potential contraindications to treatment and identify modifiable risk factors such as smoking, which is linked with an increase in oestrogen metabolism leading to vaginal atrophy^8^. A sexual history should be taken not only to establish if the woman suffers from vaginal dryness and dyspareunia—the most common and bothersome symptoms of GSM^9,10^—but also to determine the presence of sexual dysfunction. Women should be educated regarding the benefits of physical therapy, particularly in the presence of high-tone or non-relaxing pelvic floor muscle dysfunction that is triggered by painful sexual activity related to GSM^11^. Furthermore, women should be advised that it is safe and beneficial to continue with sexual activity and that there is a positive link between the lubricative response to sexual arousal and the maintenance of vaginal elasticity^12^. Masturbation or the use of lubricated sex devices are options for women without a partner^13,14^.

### Lubricants

**Figure 1.**
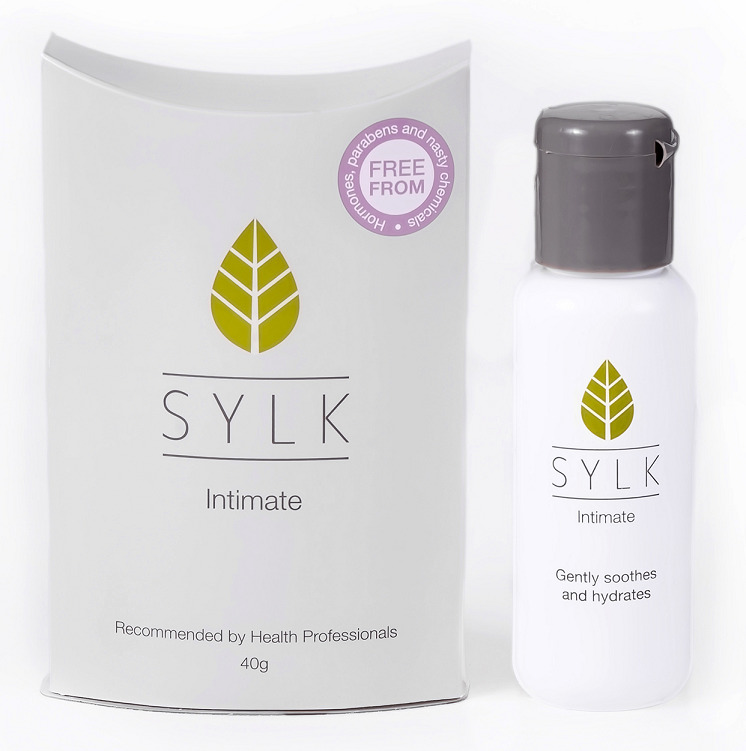
SYLK Lubricant and Moisturiser®. This figure was provided by SYLK (www.sylk.co.uk) with copyright permission to publish in this manuscript.

**Figure 2.**
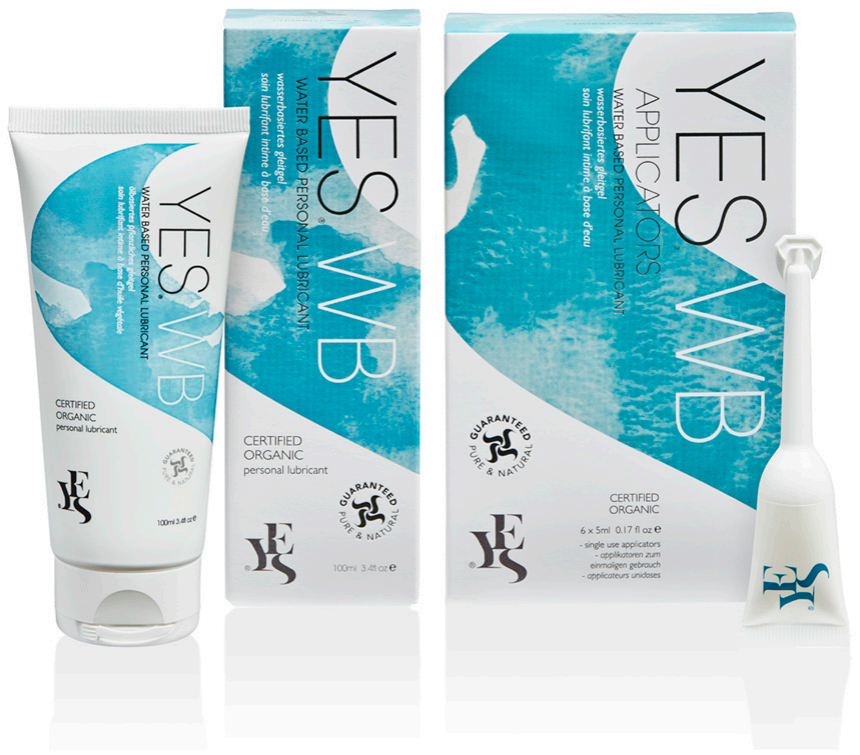
YES Water-Based Lubricant®. This figure was provided by The YES YES Company Ltd (www.yesyesyes.org) with copyright permission to publish in this manuscript.

The first-line treatment for the symptoms of vaginal dryness and dyspareunia is the use of a non-hormonal vaginal lubricant during intercourse and the regular use of a long-acting vaginal moisturiser. They can be used alone or in combination with oestrogens^15^. Lubricants are fast-acting, provide temporary relief, and are particularly beneficial for women whose symptoms primarily occur with coitus. Lubricants are available in a variety of preparations; they can be water, silicone, or oil based. Water-based lubricants have the advantage of being non-staining; women should be advised that some oil-based lubricants should be avoided, as they risk latex condom breakdown, potentially placing women at risk of sexually transmitted infections^16^. Lubricants differ to moisturisers in that they are not absorbed into the skin. Lubricants are specifically designed to reduce friction-related irritation to atrophic tissues during coitus, increasing comfort and reducing dyspareunia^17^. Examples of vaginal lubricants include Sylk Lubricant and Moisturiser (Figure 1) and YES Water-Based Lubricant (Figure 2).

### Moisturisers

**Figure 3.**
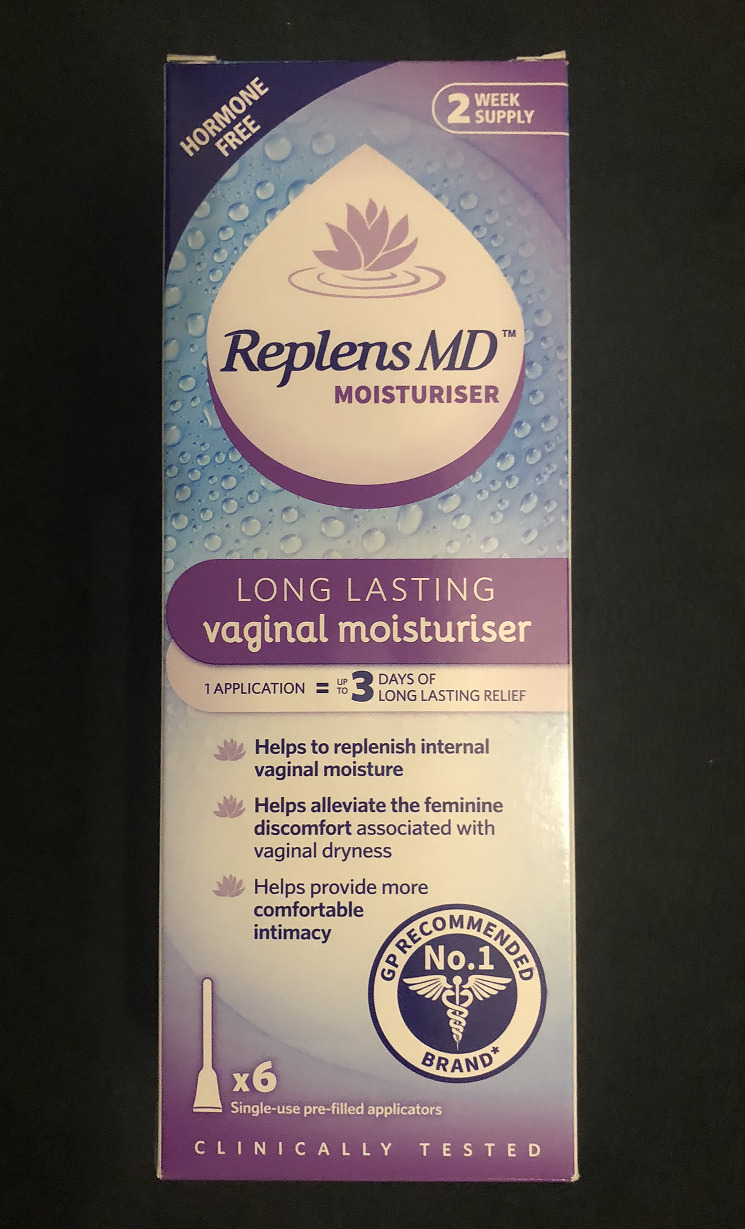
Replens MD Moisturiser®. The authors took this photograph of the product for this manuscript.

**Figure 4.**
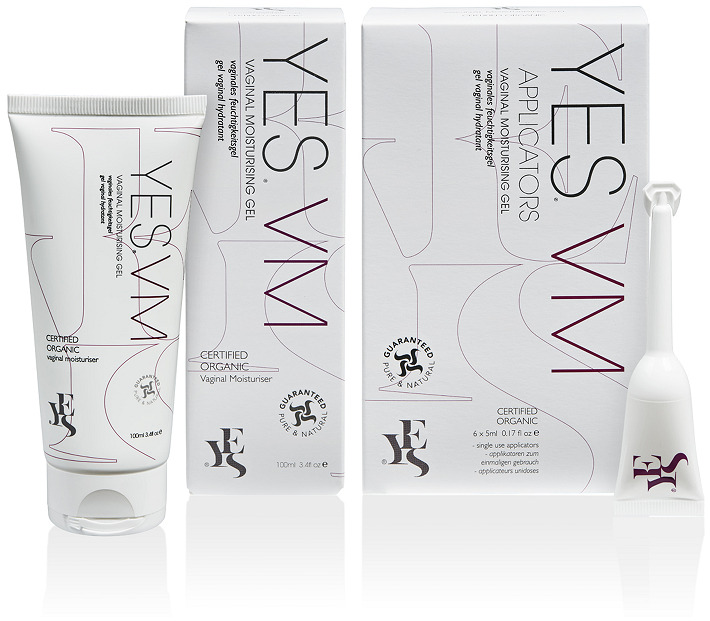
YES VM Vaginal Moisturising Gel ®. This figure was provided by The YES YES Company Ltd (www.yesyesyes.org) with copyright permission to publish in this manuscript.

Vaginal moisturisers are an excellent option for women who experience ongoing discomfort due to vaginal dryness. Moisturisers are hydrophobic and have bio-adhesive properties. They are absorbed into the skin and adhere to the superficial cells of the vagina. They have the ability to retain moisture, which is then released locally, mimicking physiological vaginal secretions. Moisturiser can be used several times per week independent of sexual activity^16^. Moisturisers contain a variety of excipients targeted at preserving pH and moderating viscosity. In a recent meta-analysis of randomised controlled trials (RCTs) comparing moisturisers and lubricants to vaginal oestrogen, dyspareunia significantly improved with both treatments, with vaginal oestrogens demonstrating superior efficacy overall, although the authors concluded that the overall data were too limited owing to small sample sizes to make a definitive conclusion^18^. An example of a vaginal moisturiser is Replens MD Moisturiser (Figure 3) and YES VM Vaginal Moisturising Gel (Figure 4).

There are multiple products available over the counter with limited research comparing them, which can be confusing for the consumer and physician. Women should be advised to choose a product that is optimally balanced in terms of pH and osmolality so that it is physiologically more similar to natural vaginal secretions. The World Health Organisation (WHO) has proposed guidance on the recommended osmolality and pH of lubricants^19^. Moisturisers and lubricants are useful for women who have a genuine contraindication to oestrogens, such as breast cancer patients on aromatase inhibitors.

### Hyaluronic acid

Hyaluronic acid (HA) has been widely used as an essential ingredient in topical hydrating and lubricating gels and has been injected for conditions such as dyspareunia. It is a naturally occurring polysaccharide and is one of the main components of the extracellular matrix present in the epithelium of many tissues, including the vagina. It has strong antioxidant properties that connect water to tissue and is considered a naturally occurring reservoir of body water that can increase moisture levels within cells and improve atrophic symptoms^20^. Various prospective observational studies carried out on HA have shown that this compound is well tolerated without side effects among patients^21^. A recent systematic review comparing the efficacy of HA and oestrogen on atrophic vaginitis showed that all articles reported improvement with both treatments favouring oestrogen. However, studies were of poor quality, small sample size, and insufficient number to reach a conclusion^22^.

**Figure 5.**
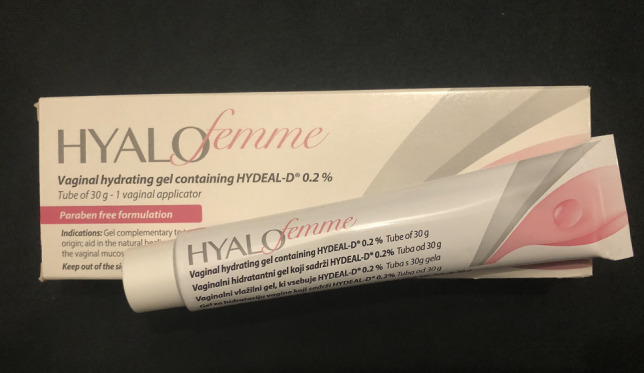
Hyalo Femme, Vaginal hydrating gel containing HYDEAL-D® 0.2%. The authors took this photograph of the product for this manuscript.

Although some women achieve improvement of symptoms from a good-quality, non-hormonal product^23^, it is essential that women are advised about the endocrinological aetiology surrounding their symptoms; GSM is a progressive and chronic condition, and without the replacement of sex steroids, long-term resolution is unlikely to occur. An example of hydrating gel is Hyalo femme (Figure 5) which contains HYDEAL-D 0.2% (an ester of hyaluronic acid).

### Laser

**Figure 6.**
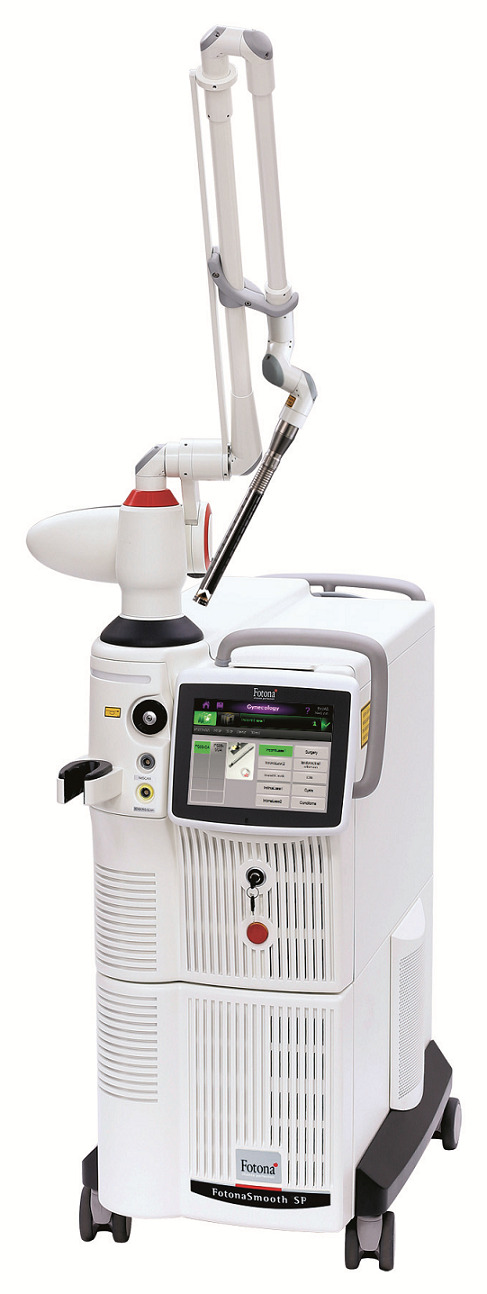
Er:YAG, Fotona Smooth SP. This figure was provided by Fotona (www.fotona.com) with copyright permission to publish in this manuscript.

**Figure 7.**
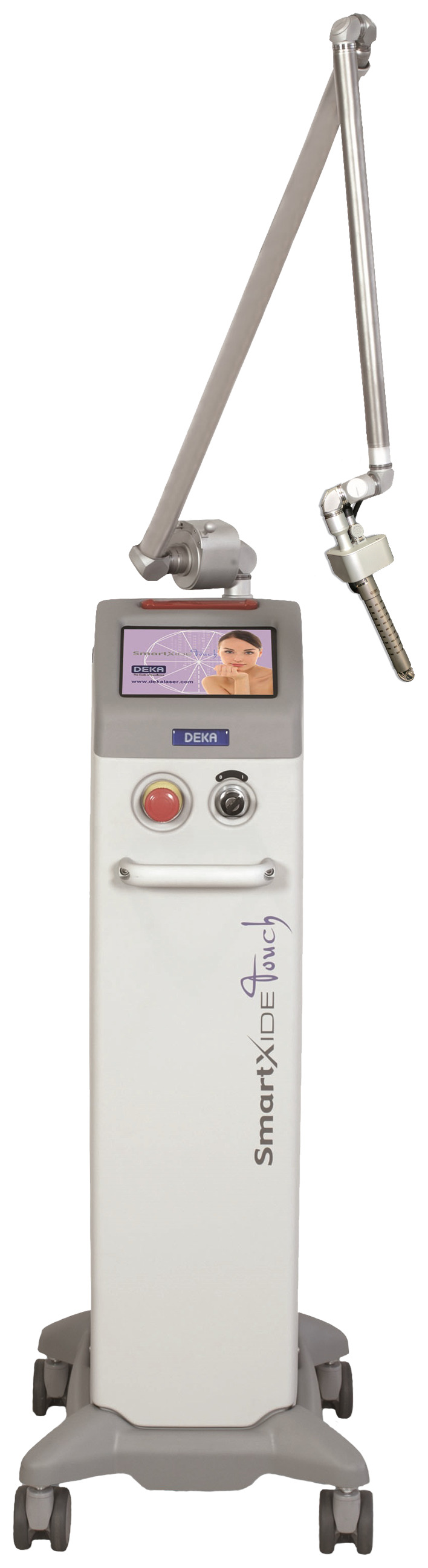
CO_2_ Laser (Mona Lisa Touch, DEXA). This figure was provided by Cynosure (www.cynosureuk.com) with copyright permission to publish in this manuscript.

Light amplification by stimulated emission of radiation (laser) was first described over a century ago and has been used in various aspects of medicine since then. Following the seminal study by Salvatore *et al*.^24^, the use of vaginal laser has grown in popularity as an alternative non-hormonal treatment for GSM. In the UK to date, laser has been used in the private sector, where it is very expensive, or for research purposes. The two most commonly used laser technologies are non-ablative photothermal Erbium:YAG laser (Er:YAG laser) and CO_2 _laser^25^ (Figure 6 and Figure 7). Through thermomodulation, these laser technologies result in the restoration of the vaginal epithelium to a state similar to that of a pre-menopausal woman^26^. The controlled temperature rise causes collagen remodelling and synthesis, neovascularisation, vasodilatation, and elastin formation^27–29^.

The safety profile surrounding energy-based devices is an area where robust research is lacking. In July 2018, the US Food and Drug Administration (FDA) issued a warning to healthcare professionals declaring that the safety and effectiveness of these devices for vaginal rejuvenation or cosmetic vaginal procedures are not proven. In addition, it stated that no energy-based device had been approved for these procedures or the treatment of menopause-related vaginal symptoms, urinary incontinence, or sexual function. As a result of this, several international professional bodies have released consensus statements, with the majority reporting that the routine use of laser should not be recommended and that further research is needed. There are several complications reported, with the most common being procedure-related pain; other reported complications include numbness, burning, dyspareunia, bladder disturbance, worsening symptoms, scarring, and worsening of lichen sclerosis^30^. Data are lacking to quantify these complications.

Systematic reviews of the literature have shown an overall improvement in the Vaginal Health Index (VHI) score and in subjective symptoms of GSM in the short term; however, studies were often non-randomised, lacked placebo/sham, and had a small sample size and follow-up duration^25,31^. This is an emerging area, and although early results appear promising, robust randomised controlled studies are required to determine the true safety and efficacy of this treatment modality.

## Pharmacological treatment

### Local oestrogens

**Figure 8.**
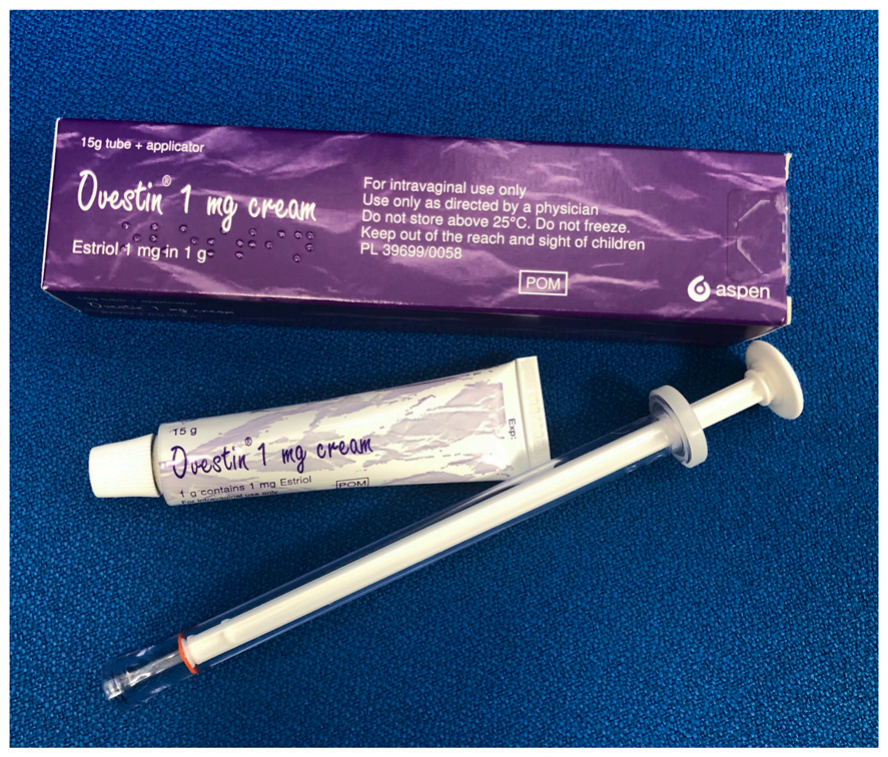
Ovestin ® oestriol vaginal cream. The authors took this photograph of the product for this manuscript.

Local vaginal oestrogen has been the treatment of choice for decades for the management of postmenopausal women with vulvovaginal symptoms of the menopause only. It allows for a lower dose of oestrogen than that used in systemic therapy for VMS^32^. A third of women on systemic hormonal replacement therapy (HRT) also have symptoms of GSM and require additional local oestrogens. Intravaginal oestrogen therapy is available in a variety of different oestrogen compounds, doses, and routes of administration, including oestriol (cream [Ovestin®] and pessary [Orthogynest®]), oestradiol (tablets [Vagifem®] and ring [Estring®]), and a conjugated preparation (cream [Premarin®])^33^ (Figure 8–Figure 10). The choice among different local oestrogen treatments depends on the severity of symptoms and the patient’s preference^4^.

**Figure 9.**
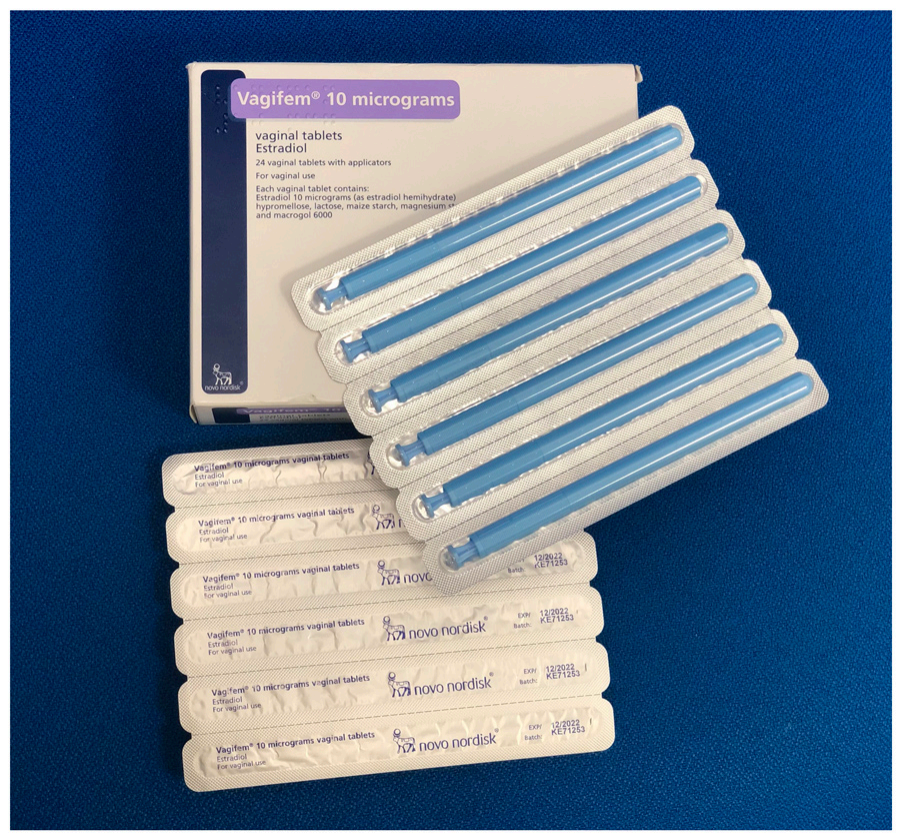
Vagifem® oestradiol vaginal pessary. The authors took this photograph of the product for this manuscript.

**Figure 10.**
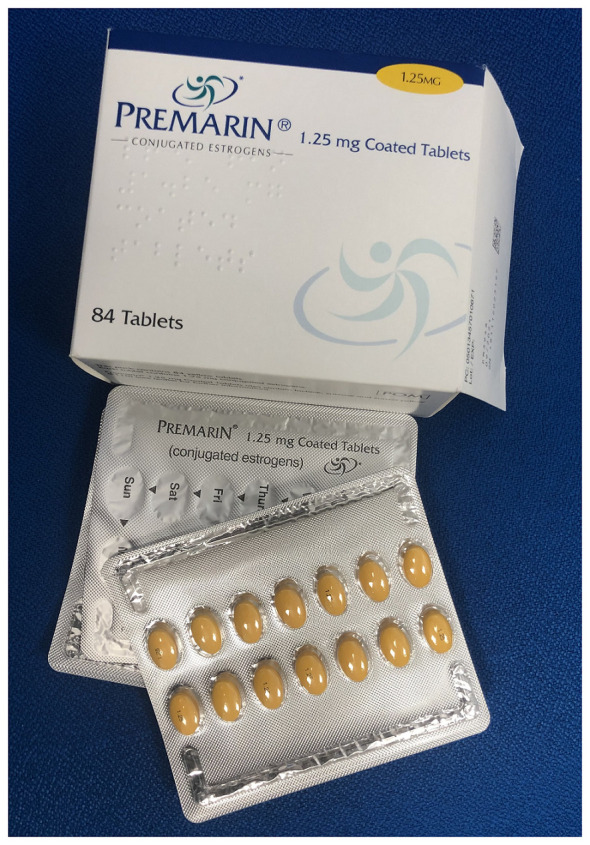
Premarin® conjugated oestrogen vaginal cream. The authors took this photograph of the product for this manuscript.

The typical administration schedule, with the exception of the oestradiol ring, which is used continuously for 90 days, consists of an initial loading dose of daily applications followed by a maintenance regimen for as long as it is needed to manage symptoms, usually indefinitely. Ovestin® is applied daily for 3 weeks, then three times a week. Vagifem® is an intravaginal tablet pessary that is administered daily for 2 weeks, followed by a twice-weekly maintenance schedule^4^. Systemic absorption has been shown to occur with Premarin vaginal cream and is not entirely safe in women who retain a uterus owing to the unopposed oestrogen hyperplastic effect on the endometrium^34^. Premarin® has been withdrawn in the UK but is still available in other countries^4^.

The rationale behind this administration regimen is that the absorption of oestrogens is highest during the first few days of treatment when the vaginal epithelium is most atrophic, has increased vascularity, and has a thinner superficial epithelial layer. Once the epithelium has matured, the absorption of local oestrogen decreases and therefore smaller doses of oestrogen are sufficient to prevent recurring atrophy while ensuring the systemic absorption from chronic use of oestrogen is minimised^35^. The safety and efficacy of these regimens were supported by two large randomised, double-blind, placebo-controlled trials from the same working group. The authors identified that the administration schedule of daily application of an oestradiol vaginal cream at 0.003% for 2 weeks then two or three times a week for women with the principle bothersome symptom of vaginal dryness or dyspareunia was effective and well tolerated^36,37^.

Systematic efficacy and safety reviews of vaginal oestrogen products for the treatment of moderate-to-severe GSM have reported them to be superior to placebo in achieving subjective improvement in vaginal dryness, dyspareunia, and urogenital symptoms. In addition, objective measures of improvement were found in gross vaginal epithelial appearance, maturation of vaginal epithelium, reduction in pH, and increased vaginal *Lactobacillus*^38–40^. The latest Cochrane review on local oestrogen for vaginal atrophy in postmenopausal women included 30 clinical studies with 6,235 postmenopausal women. It compared different vaginal oestrogen preparations with each other and placebo. The authors concluded that all compounds improved symptoms of VVA in comparison to placebo with minimal safety concerns. There were no significant differences in efficacy among the different preparations^41^.

The systemic absorption of oestrogens remains the primary concern surrounding the use of local oestrogens. While the local route of administration minimises the exposure, it is not entirely eliminated. Local oestrogen is associated with an acute rise in plasma oestradiol levels, with a peak at approximately 8 hours and a return to baseline at 12 hours, never rising again after that or when vaginal oestrogen is stopped and later restarted^35^. This sharp rise in plasma oestradiol level may be the cause of some systemic adverse effects reported, such as vaginal bleeding or breast tension. Oestradiol tablets and ring are associated with systemic absorption that is equal to or less than that produced by the postmenopausal adrenal gland^35^.

Oestriol cream is associated with greater systemic absorption; however, as oestriol is a weak oestrogen that is not converted to oestradiol, the systemic effects are believed to be reduced^4^. The higher absorption level encountered with oestriol cream may be advantageous for women with more severe symptoms; however, some women complain that creams are cumbersome, messy to insert, and associated with greater vaginal discharge compared with the tablet pessary and rings^42^. The phase 3 trial REJOICE investigated the recently FDA-approved 17β-oestradiol softgel vaginal insert. It identified significantly improved dyspareunia and vaginal dryness compared to placebo, with improvements observed as early as week 2 with most doses^43^. Furthermore, in the review of efficacy and pharmacokinetic data of the same study, the systemic absorption of oestradiol was minimal^44^. These are promising early results for this new preparation.

A low dose of topical vaginal oestrogen is as effective as systemic oestrogen therapy for the treatment of GSM^45^ with similar improvement seen with all compounds, although efficacious local oestrogens are not an appropriate treatment for all women with GSM. If there are contraindications, difficulties in use, or use of hormonal treatment unacceptable to the user, other therapeutic options should be explored.

### Consideration in women with breast cancer

More than 60% of postmenopausal women with breast cancer report symptoms of GSM^46^. Management of these women, particularly those with oestrogen receptor (ER) positive breast cancer, poses a challenge. These women often experience profound symptoms of GSM, but treatment options are limited. Women at high risk for breast cancer or those with ER-positive breast cancers who are taking tamoxifen with persistent or severe symptoms not responding to non-hormonal therapies may be offered low-dose vaginal oestrogen therapies, provided they have factors indicating a low risk of recurrence^47^. Most oncologists are opposed to the use of even vaginal oestrogen in women taking aromatase inhibitors; however, if symptoms are profound following discussion with their oncologist and there is recognition that even a small amount of oestrogen absorbed may impact the effectiveness of the aromatase inhibitor on some occasions, low-dose vaginal hormone therapy may be considered^4^. The use of vaginal oestrogen in women with a history of triple-negative disease is theoretically reasonable, but data are lacking.

### Systemic hormonal replacement therapy

Systemic oestrogen therapy is the mainstay of treatment for VMS of the menopause and can have a beneficial effect in improving concurrent symptoms of GSM. However, systemic HRT for GSM alone is not FDA approved or NICE recommended but can be considered in the treatment of concurrent VMS and GSM symptoms^48,49^. Oestrogen alone can be used for hysterectomised women but needs to be combined with progestogen therapy for women with an intact uterus. Although systemic HRT can be beneficial in improving symptoms of GSM, up to 25% of women will still experience symptoms of urogenital atrophy^50^. Furthermore, it is essential that when VMS subside and symptoms are limited to GSM, systemic treatment is discontinued, and local vaginal treatment alone is recommended. Systemic HRT can increase the risk of breast cancer and thromboembolic disease; therefore, a benefit/risk assessment needs to be followed^48,49^.

### Prasterone (dehydroepiandrosterone)

There are three major naturally occurring oestrogens in women; oestrone (E1), oestradiol (E2), and oestriol (E3). In pre-menopausal women, the most abundant oestrogen is oestradiol, which is produced from the granulosa cells of the ovary; in addition, androgen production is significantly greater than that of oestrogens^51^. At menopause, the secretion of oestradiol by the ovaries stops and dehydroepiandrosterone (DHEA) becomes the exclusive precursor for all sex steroids made intracellularly in peripheral tissues independent of the ovary^52^. Sex steroid activity becomes exclusively dependent upon the ability of each tissue to transform DHEA into oestrogens and androgens for local and intracellular use^53^.

**Figure 11.**
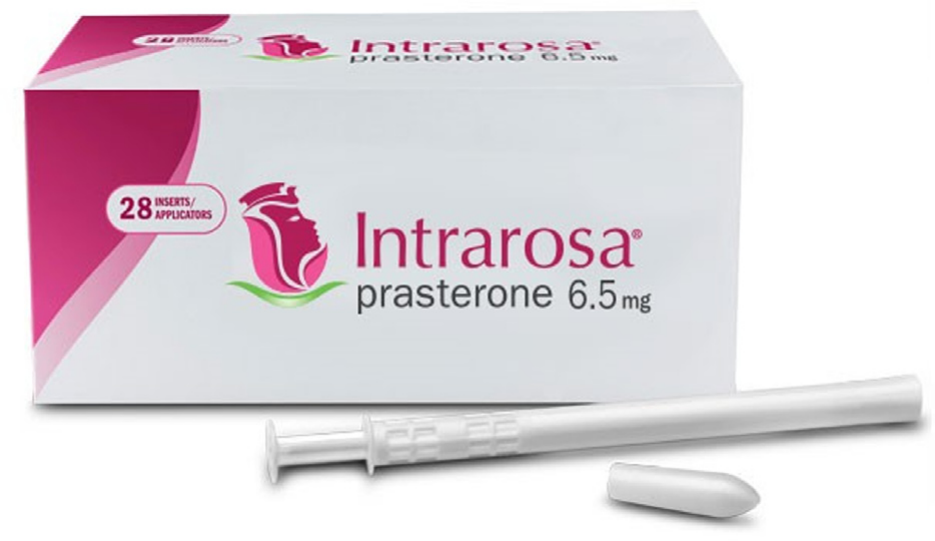
Intrarosa ® prasterone. This figure was provided by Theramex (www.theramex.com) with copyright permission to publish in this manuscript.

Androgen receptors are ubiquitous in the genitourinary tract^51^. Together with that of oestrogens, the level of androgens falls with increasing age, potentially contributing to the symptoms and signs of GSM^54^. Prasterone (Intrarosa®) is a synthetic form of endogenous DHEA and is licensed for the treatment of moderate-to-severe dyspareunia (Figure 11). It exerts trophic effects on various genitourinary tissues, transforming DHEA into the appropriate amount of oestrogens and androgens strictly for intracellular and local action without biologically significant changes in serum sex steroids occurring. Crucially, serum levels of all sex steroids remain within normal values with intravaginal DHEA^55^.

Prasterone is administered as a vaginal insert once daily at bedtime^33^. When applied intravaginally, prasterone is converted not only into oestrogens but also into androgens such as testosterone, androstenedione, and dihydrotestosterone (DHT). Studies have shown that the use of prasterone when compared to placebo is an effective treatment for vaginal dryness, burning, itching, and global sexual symptoms with increased libido and lessening of dyspareunia. Objective parameters, namely acidity of vaginal pH, thickening of epithelium, and vaginal maturation index (VMI), also improved compared to baseline and placebo^56^. Pre-clinical studies of prasterone found improvement of the collagen and muscularis layers of vaginal tissues and increased nerve density in the vagina^57^. Although serum oestradiol and testosterone levels do not seem to increase after intravaginal prasterone, further studies are needed to examine its safety in patients with a history of hormonal cancers.

### Selective oestrogen receptor modulators (ospemifene)

The concerns issued regarding hormonal treatments and the potential stimulating effects of systemic oestrogens in breast and endometrial tissue have prompted the development of alternative treatments. Selective oestrogen receptor modulators (SERMs) are synthetic non-steroidal agents that have the potential to exert a variable agonistic, antagonistic, or neutral effect on oestrogen receptors in targeted tissues. Examples include ospemifene, lasofoxifene, raloxifene, bazedoxifene, and tamoxifen. Along with ospemifene, lasofoxifene has demonstrated a positive impact on vaginal tissue in postmenopausal women, and although several studies have found that lasofoxifene results in significant improvement in objective parameters of vaginal pH and VMI, the clinical development of this SERM is on hold^58^. Raloxifene and tamoxifen are ineffective, as they lack the oestrogenic agonist effect in urogenital tissue. Bazedoxifene is a newer SERM that preferentially blocks oestrogen action in the endometrium and uterus compared with other available SERMs. In the Selective Estrogen Menopause and Response to Therapy (SMART) trials^59^, vaginal maturation index improved, as did ease of lubrication and sexual function compared with placebo in postmenopausal women with VVA^59^. Conjugated equine oestrogen/bazedoxifene is labelled and FDA approved for the treatment of moderate-to-severe VMS associated with menopause and prevention of postmenopausal osteoporosis. VVA is not an FDA-approved indication. Ospemifene is the only SERM currently approved for the treatment of moderate-to-severe dyspareunia and vaginal dryness, although the European Medical Agency (EMA) has recommended that treatment is reserved for those who are not eligible for local vaginal oestrogen therapy, a restriction not imposed by the FDA^60^.

**Figure 12.**
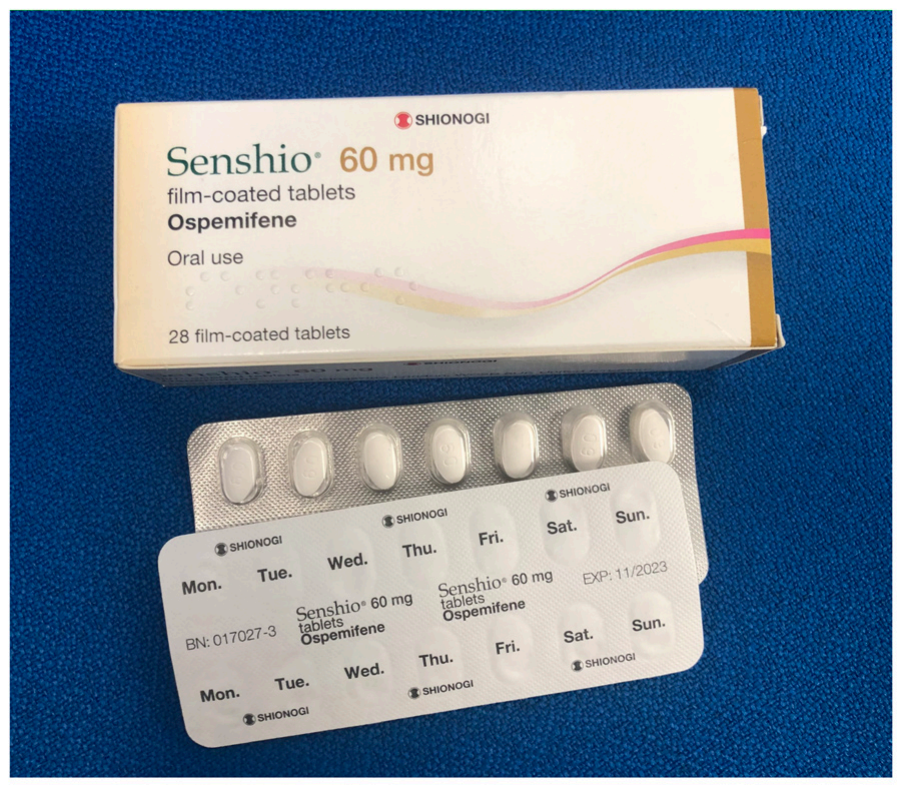
Osphena ® Ospemifena tablets. The authors took this photograph of the product for this manuscript.

Ospemifene (Osphena®) is the only approved orally administrated non-hormonal treatment for GSM^33^ (Figure 12). Various studies have found that, compared to placebo, it leads to a significant increase in superficial cells, reduced parabasal cells, thickening of the vaginal epithelium, and lowering of pH to a normal level^61–63^. The effects of ospemifene are seen from the fourth week of treatment and endure for up to 1 year. A recent prospective study based on vulvoscopic photographs taken at screening and on completion of a 20-week course of ospemifene therapy identified significant changes in introital stenosis, urethral meatal prominence, vestibular pallor, mucosal moisture, vestibular erythema, vaginal rugae, and anterior wall prominence^64^. In addition, ospemifene has demonstrated that it may be beneficial for urinary and sexual symptoms associated with GSM. In a study of over 100 postmenopausal women with moderate-to-severe GSM, symptoms of overactive bladder, stress incontinence, and sexual function improved^65^. When compared to local vaginal oestrogen therapy in an indirect comparison systematic review, ospemifene 60 g once per day was found to be as, if not more, well tolerated, safe, and effective as vaginal oestrogens^66^.

Ospemifene is generally well tolerated; the most common treatment-emergent adverse events reported include hot flushes, vulvovaginal candidiasis and other mycotic (fungal) infections, headaches, muscle spasm, vaginal discharge, vaginal bleeding, and rash^64,67^. Contraindications to ospemifene include women with undiagnosed postmenopausal vaginal bleeding and known or suspected oestrogen-dependent neoplasia currently undergoing treatment^62,68^. Caution is suggested for patients with active or a history of venous thromboembolism (VTE), with the EMA advising individualised care^69^, although a recent review of pharmacovigilance reporting data of potential risks at the 2-year post-approval review indicated that the incidence of VTE was lower for ospemifene treatment than the identified background risks.

## Conclusion

Patient preference is paramount, as adherence and compliance are crucial in obtaining a significant improvement in GSM symptoms and quality of life. The principal goal when treating GSM is to relieve symptoms but cost and treatment safety need to be considered for each individual. Low-dose vaginal oestrogen in the form of either Vagifem® or Ovestin®, when not contraindicated, remains the most effective and economical option and has the fewest side effects. However, there are some women who do not wish to use oestrogen because of personal or cultural reasons and others in whom oestrogen is genuinely contraindicated. In these circumstances, the development of novel efficacious and safe interventions is essential to expand our armature for such. To date, whilst there are many other available treatments, each with benefits and limitations, there are very few comparative studies that can recommend one treatment over another. Safety and efficacy need to be considered in every case, and a discussion should be undertaken to ascertain the individual’s preferences and concerns; otherwise, compliance will be poor.
